# Physical Determinants of Vitamin D Photosynthesis: A Review

**DOI:** 10.1002/jbm4.10460

**Published:** 2021-01-19

**Authors:** Jonathan J Neville, Tommaso Palmieri, Antony R Young

**Affiliations:** ^1^ St John's Institute of Dermatology, School of Basic & Medical Biosciences King's College London London United Kingdom

**Keywords:** ACTION SPECTRUM, ENVIRONMENT, SUNSCREEN, ULTRAVIOLET RADIATION (UVR) DOSE, VITAMIN D

## Abstract

Vitamin D synthesis by exposure of skin to solar ultraviolet radiation (UVR) provides the majority of this hormone that is essential for bone development and maintenance but may be important for many other health outcomes. This process, which is the only well‐established benefit of solar UVR exposure, depends on many factors including genetics, age, health, and behavior. However, the most important factor is the quantity and quality of UVR reaching the skin. Vitamin D synthesis specifically requires ultraviolet B (UVB) radiation that is the minority component (<5%) of solar UVR. This waveband is also the most important for the adverse effects of solar exposure. The most obvious of which is sunburn (erythema), but UVB is also the main cause of DNA damage to the skin that is a prerequisite for most skin cancers. UVB at the Earth's surface depends on many physical and temporal factors such as latitude, altitude, season, and weather. Personal, cultural, and behavioral factors are also important. These include skin melanin, clothing, body surface area exposed, holiday habits, and sunscreen use. There is considerable disagreement in the literature about the role of some of these factors, possibly because some studies have been done by researchers with little understanding of photobiology. It can be argued that vitamin D supplementation obviates the need for solar exposure, but many studies have shown little benefit from this approach for a wide range of health outcomes. There is also increasing evidence that such exposure offers health benefits independently of vitamin D: the most important of which is blood‐pressure reduction. In any case, public health advice must optimize risk versus benefit for solar exposure. It is fortunate that the individual UVB doses necessary for maintaining optimal vitamin D status are lower than those for sunburn, irrespective of skin melanin. © 2020 The Authors. *JBMR Plus* published by Wiley Periodicals LLC. on behalf of American Society for Bone and Mineral Research.

## Introduction

Terrestrial solar ultraviolet radiation (UVR; ~295–400 nm) can be divided into ultraviolet B (UVB; 280–315 nm) and ultraviolet A (UVA; 315–400 nm), the vast majority (≥95%) of which is UVA. Exposure to sunlight has many effects on human health^(^ ^1^ ^)^ including erythema (sunburn), skin cancer, and vitamin D synthesis. Until recently, vitamin D synthesis was regarded as the only benefit from solar exposure, but there is increasing evidence for other health benefits that are independent of vitamin D,^(^ ^1^, ^2^ ^)^ such as reduced blood pressure.^(^ ^3^, ^4^, ^5^ ^)^ All UVR effects are initiated by the absorption of UVR by chromophores.^(^ ^6^ ^)^ The absorption spectrum of a given chromophore determines the action spectrum (wavelength dependence) of the given photobiological outcome.

**Fig 1:**
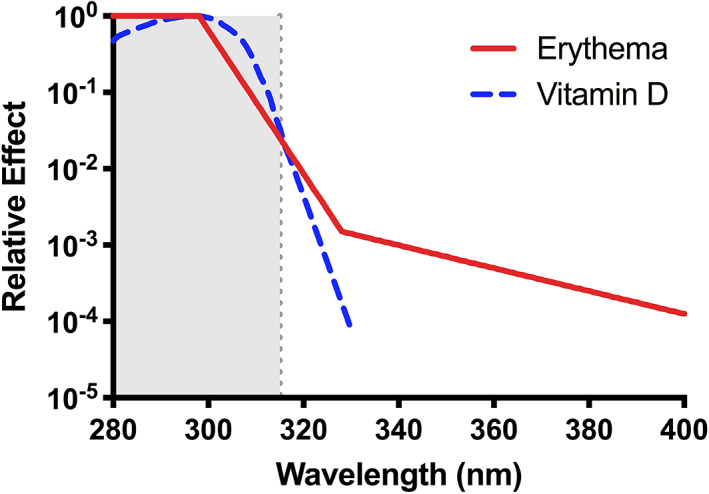
Commission Internationale de l'Eclairage (International Commission on Illumination) action spectra for erythema^(^ ^35^ ^)^ and cutaneous previtamin D_3._^(^ ^9^ ^)^ Note the considerable overlap in the solar UVB (~295–315 m) region.

Solar UVB (~295–315 nm) converts 7‐dehydrocholesterol (7‐DHC), a chromophore in epidermal keratinocytes and dermal fibroblasts, into previtamin D_3._^(^ ^7^, ^8^ ^)^ Figure 1 shows the action spectrum for this process,^(^ ^9^ ^)^ though the validity of this spectrum has been questioned.^(^ ^10^ ^)^ Previtamin D_3_ is thermally unstable and isomerizes into vitamin D_3_ (cholecalciferol).^(^ ^11^ ^)^ This is hydroxylated in the liver to 25‐hydroxyvitamin D [25(OH)D] and then in the kidneys to 1,25‐dihydroxyvitamin D [1,25(OH)_2_D], which is the active hormone. Many tissues, including skin, have the enzymes for both hydroxylations.^(^ ^12^ ^)^

Vitamin D is essential for bone health and may play a role in many diseases.^(^ ^1^ ^)^ Most 1,25(OH)_2_D (eg, 70%–85% in summer in the White population of the United Kingdom [UK]^(13^ ^)^) originates from cutaneous photosynthesis. The rest is provided by diet and supplementation: vitamin D_3_ from animal products and also from plants^(^ ^14^, ^15^ ^)^ and vitamin D_2_ (ergocalciferol) from fungi.^(^ ^15^ ^)^

Solar exposure for vitamin D synthesis must be balanced against risks: The most obvious is erythema. The minimal erythemal dose (MED) is an individualized clinical measurement of personal sensitivity to UVR; it represents the lowest UVR dose (J/m^2^) required to cause just perceivable erythema on irradiated skin. MED shows considerable interpersonal variation.^(^ ^16^ ^)^ It is also is very dependent on the UVR source used. Thus, it is approximately 250 J/m^2^ with monochromatic UVB at 300 nm, whereas it is approximately 320,000 J/m^2^ with UVA at 360 nm.^(^ ^17^ ^)^

Many non‐UVB factors influence vitamin D status including genetics, metabolism, health, and age.^(^ ^18^, ^19^, ^20^, ^21^ ^)^ Here we assess those factors that influence the quantity and quality of UVB reaching cutaneous 7‐DHC. These include spectral, atmospheric, geographic, and behavioral factors, as well as the skin's melanin content. In addition, we determine the minimal doses of erythemally effective UVR necessary for this purpose. Erythema, which typically peaks at approximately 24 hours after solar exposure, is the most widely used endpoint for risk assessment; its avoidance is advocated in public health campaigns for skin cancer prevention.

## Vitamin D Deficiency

Serum 25(OH)D concentration gives the most accurate estimate of vitamin D status.^(^ ^22^ ^)^ There are different definitions of vitamin D insufficiency/deficiency; however, serum 25(OH)D <50 nmol/L is widely used for insufficiency.^(^ ^23^ ^)^ Suboptimal vitamin D status is widespread. A study of almost 56,000 people in Europe reported that 40.4% had insufficiency according to the above definition, especially those with darker skin.^(^ ^24^ ^)^ Globally, 37.3% had 25(OH)D concentrations <50 nmol/L, and global variability in vitamin D status showed no correlation with latitude.^(^ ^25^ ^)^ A recent report showed that Africa had much poorer vitamin D status than other parts of the world: with 34% with 25(OH)D <50 nmol/L^(^ ^26^ ^)^ but there are exceptions as discussed in the Skin Pigmentation subsection. However, there is a lack of data from Africa and South America, and for infants, children, adolescents, and pregnant women worldwide.^(^ ^27^, ^28^ ^)^ The authors of a 2007 article reported that 46.6% of White UK adults (aged 45 years) had 25(OH)D <40 nmol/L in winter/spring, which improved to 15.4% of these adults in summer/autumn. The odds ratio for increased risk was 2.03 for obesity and 2.38 for living in Scotland (compared with southern England).^(^ ^29^ ^)^ More recent studies have reported 23% and 61% of UK adults (19–64 years) with serum 25(OH)D <25 nmol/L and <50 nmol/L, respectively.^(^ ^30^ ^)^ A large sample of UK South Asians (aged 40–69 years) showed 92% with serum 25(OH)D <50 nmol/L, 55% <25 nmol/L, and 20% <15 nmol/L.^(^ ^31^ ^)^ A study of 5034 Australian adults reported that 20% of participants had serum 25(OH)D <50 nmol/L.^(^ ^32^ ^)^ Newborns and the elderly living in institutions are at greatest risk of deficiency.^(^ ^25^ ^)^

## UVR Spectral Factors

Irradiance and action spectra are critical considerations in photobiological research and its public health consequences. Incorrect conclusions can be reached without a good definition of these spectra and their interactions.

### Irradiance spectrum

An irradiance spectrum is a plot of UVR intensity received per unit of area (measured as W/m^2^/nm) versus wavelength. The integral of this plot is expressed as W/m^2^. At the Earth's surface, the solar irradiance spectrum has a dynamic range of six orders of magnitude. Even the weaker spectral subranges may have profound biological effects (see below). Therefore, the irradiance spectrum should be measured with an instrument (spectroradiometer) that can accurately handle six orders of magnitude.

**Fig 2:**
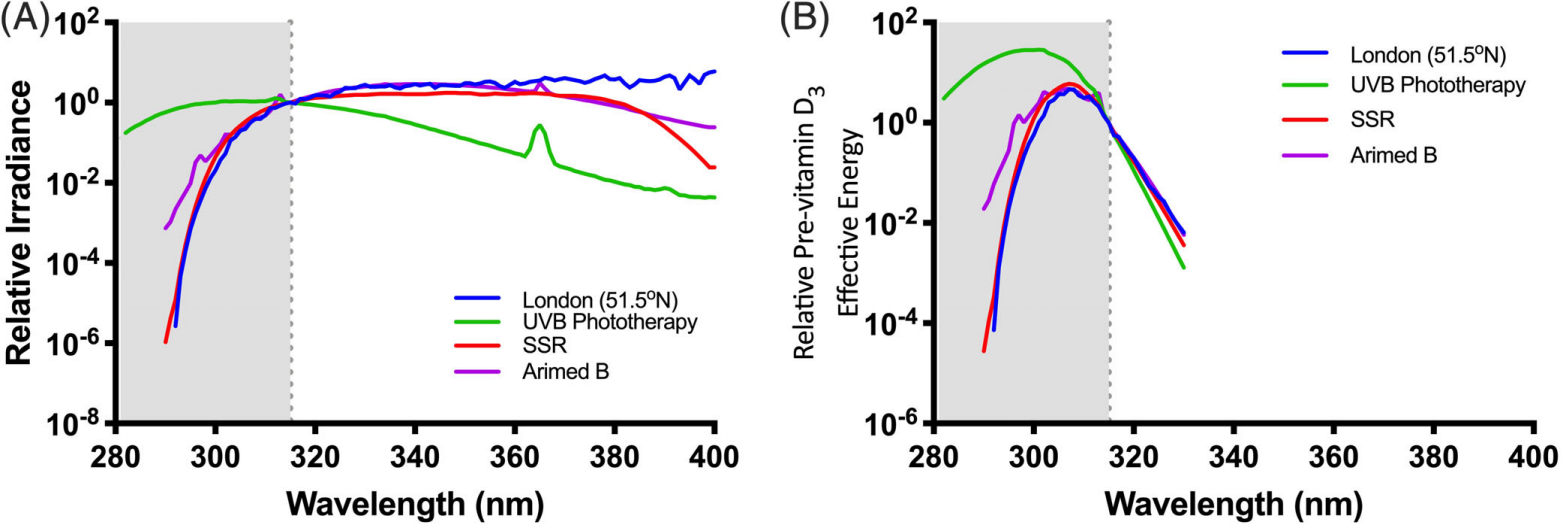
(*A*) Relative irradiance spectra of London (United Kingdom) noon midday midsummer solar UVR and xenon arc SSR as used to test sunscreens, fluorescent SSR (Arimed B), and a broad spectrum UVB phototherapy source. Note greater shorter nonsolar wavelength UVB content in phototherapy spectrum compared with sunlight (*B*). The use of the CIE action spectrum for previtamin D_3_ (Fig. 1) as a weighting function for the spectra is shown in (*A*). Note the large disproportionate effect of the nonsolar UVB with the phototherapy source. Graphs normalized at 315 nm, which is the end of shaded area. SSR = solar simulating radiation; UVB = ultraviolet B; UVR = ultraviolet radiation.

A solar UVR irradiance spectrum is shown in Fig. 2*A*. Many vitamin D studies have been done with broadband UVB phototherapy sources. However, as seen in Fig. 2*A*, these typically emit nonsolar UVB (wavelengths <295 nm) that are very effective at previtamin D_3_ production (Fig. 1) and can therefore give misleading results if used as a surrogate for solar UVR. Studies have also been done with a narrow band phototherapy source that is essentially monochromatic: approximately 313‐nm UVB. Tanning cabinets have also been used. Cutaneous production of vitamin D involves a complex set of photochemical reactions, of which the conversion of 7‐DHC to previtamin D_3_ is only one. All reactions have their own preferential wavelengths.^(^ ^33^ ^)^ Therefore, ideally, solar UVR should be used but that presents considerable logistical challenges. The best laboratory option is solar‐simulated radiation (SSR) obtained from a filtered xenon arc source. Most SSR sources are designed for sunscreen testing with very small irradiation fields. It is possible to get a good solar UVR simulation with fluorescent tubes (Fig. 2*A*).

### Action spectrum

Action spectrosccopy determines the wavelength dependence of a given photobiological outcome. This has two major purposes: (i) identification of a chromophore and (ii) the generation of a biological (or chemical) weighting function, which is called an action spectrum. An action spectrum for a given biological effect gives the relative efficacy of dose at any wavelength when compared with dose at a reference wavelength (which should be specified—usually the wavelength of the maximum value —and for which the absolute production should be given. The maximum value is usually normalized to 1). The effective irradiance spectrum of a given UVR source for a given biological outcome is the product of its irradiance spectrum with the action spectrum for that endpoint. The surface under this curve gives the total biological efficacy, as if the UVR source emits monochromatic radiation at the reference wavelength. The modification of an irradiance spectrum with an action spectrum is termed “spectral weighting.” The importance of spectral weighting can be found in a study that showed that the 0.8% UVB (ie, 99.2% UVA) content of a sunbed UVR source caused 75% of DNA damage in human keratinocytes in vitro.^(^ ^34^ ^)^

Action spectroscopy shows that measurements of terrestrial UVR exposure as J/m^2^ have little biological value per se. Thus, it is more useful to use biologically weighted UVR exposure; this is widely done for erythema as an indicator of risk. The standard erythemal dose (SED)^(^ ^35^ ^)^ is an example that is increasingly used in epidemiology. Unlike MED, this measure is independent of personal sensitivity to UVR and the irradiance spectrum. It represents a dose of 100 J/m^2^, of any irradiance spectrum that has been weighted by the Commission Internationale de l'Eclairage (CIE; International Commission on Illumination) erythema action spectrum (Fig. 1).^(^ ^35^ ^)^ The MED for a fair‐skinned person is approximately 3 SED.^(^ ^16^ ^)^ The CIE erythema action spectrum is also the basis for calculating the publicly available UV index (UVI): an international standardized, dimensionless scale quantifying the irradiance of erythemally effective UVR.

Action spectra for erythema, epidermal DNA photodamage,^(^ ^17^ ^)^ keratinocyte cancers^(^ ^36^ ^)^ and skin photoageing^(^ ^37^ ^)^ are broadly similar. Thus, the CIE erythema action spectrum is widely used to assess risk from solar UVR exposure.

Figure 1 shows the CIE action spectrum for the conversion of 7‐DHC to previtamin D_3,_^(^ ^9^ ^)^ which is widely used in risk–benefit analyses for solar UVR exposure.^(^ ^38^, ^39^, ^40^ ^)^ Maximal activity was at 297 nm. However, concerns about study methodology have led to questions about its validity and its use in risk–benefit analyses.^(^ ^10^, ^33^, ^41^, ^42^ ^)^ For example, it is based on a single ex vivo study in which human skin (unknown body site and age) was irradiated with unspecified doses of wavebands between 255 and 320 nm.^(^ ^43^ ^)^ Figure 2*B* shows the use of this action spectrum as a weighting function for solar UVR, a broad spectrum UVB phototherapy source, and xenon arc and fluorescent SSR (Arimed B). It can be seen that the UVB phototherapy source has a disproportionate effect, especially with shorter nonsolar UVB wavelengths.

It should be stressed that the CIE spectrum only represents the initial cutaneous photochemical step in the formation of vitamin D. It does not necessarily represent serum 25‐hydroxyvitamin D_3_ [25(OH)D_3_] because it does not consider other inevitably associated photochemical modifications, such as photo‐isomerization of excess previtamin D_3_ and vitamin D_3_ into tachysterol, lumisterol, suprasterol I and II, and 5,6‐transvitamin D_3_. These degradation processes may prevent vitamin D toxicity^(^ ^11^ ^)^ that may occur with supplementation^(^ ^44^ ^)^ but not with UVR exposure. If all photochemical processes are accounted for, then it can be shown that the action spectrum changes during the course of UVR exposure.^(^ ^33^ ^)^ For this reason, the use of the single CIE action spectrum to predict vitamin D production can overestimate in vivo vitamin D photosynthesis.^(^ ^33^ ^)^

Photodegradation by UVA is supported in one human study that used UVA, UVB, and mixed UVA and UVB exposures.^(^ ^45^ ^)^ The authors reported no difference in the increase of 25(OH)D between participants exposed to UVB and mixed UVA and UVB for periods of <9 minutes. Exposure for >9 minutes resulted in a significantly lower increase in 25(OH)D in the group exposed to mixed UVB and UVA compared with UVB alone. The findings of the study have, however, been called into question based on uncertainties in dosimetry.^(^ ^46^, ^47^ ^)^

It should be noted that there are other less well‐established in vitro action spectra for previtamin^(^ ^48^ ^)^/vitamin D_3_^(^ ^49^ ^)^ (cholecalciferol) formation that have been used as weighting functions for human studies and compared with the CIE action spectrum.^(^ ^42^ ^)^ Different results have been obtained with different UVR spectra. A recent in vivo human study (unpublished, Young and colleagues) suggests that the CIE previtamin D action spectrum requires a 5‐nm shift to the shorter wavelengths to be applicable for serum 25(OH)D_3_. Furthermore, this study, done with suberythemal exposures, showed no significant spectral interaction.

An action spectrum, determined with well‐defined light‐emitting diode irradiance spectra, for vitamin D_3_ in pig skin showed a peak at 296 nm when tested at two UVR doses.^(^ ^50^ ^)^ It should be noted that basing an action spectrum on a value at a predetermined dose is only valid if the dose‐response curves for all wavelengths have the same slope, otherwise the action spectrum will vary with dose.

### UVR Dose

**Fig 3:**
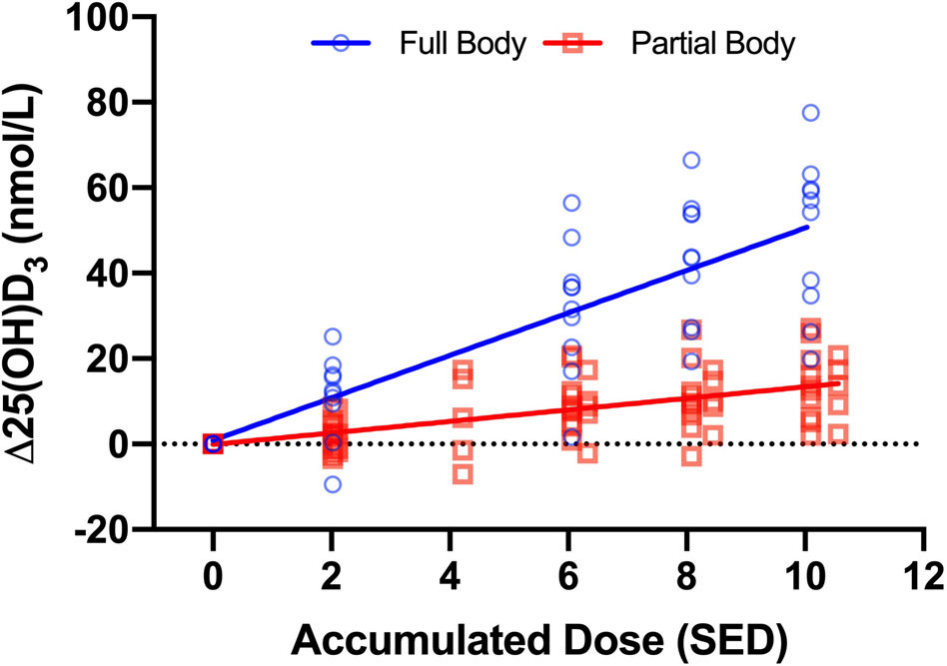
Effect of UVR dose and body surface area exposed with the same ultraviolet B phototherapy source (UV6 tubes from Waldmann GmbH & Co, Villingen‐Schwenningen, Germany). Full body (FB ~85% BSA) with *n* = 10 and partial body (PB ~4% BSA) with *n* = 15. Each site was given four to five suberythemal exposures of approximately 2 SED with intervals of 3 to 4 days, resulting in a cumulative exposure of approximately 10 SED. Increasing the exposed BSA over 20‐fold resulted in a 3.7‐fold steeper slope, which shows a nonproportional relationship between % BSA exposed and Δ25(OH)D_3_ (25‐hydroxyvitamin D_3_). Linear regression equations for FB are y = 4.97x + 0.86 (ie, 4.97 nmol/L SED), *p* = 1.78 × 10^−13^, *R* ^2^ = 0.68, and for PB are y = 1.35x − 0.09 (ie, 1.35 nmol/L SED), *p* = 1.67 × 10^−10^, *R* ^2^ = 0.41. The FB and PB slopes are significantly different: *p* = 2.33 × 10^−32^. Data from Young and colleagues (unpublished). 25(OH)D_3_ = 25‐hydroxyvitamin D_3_; SED = standard erythemal dose.

UVR dose (J/m^2^) is the product of irradiance and exposure time in seconds. Studies in humans and pigs have reported that UVR exposure and markers of vitamin D concentration show an initial linear dose‐dependent relationship that plateaus after repeated exposures.^(^ ^50^, ^51^, ^52^, ^53^, ^54^, ^55^, ^56^ ^)^ Figure 3 shows linear dose responses in healthy volunteers exposed to a broadband UVB phototherapy source. A large observational study showed a very steep rise in 25(OH)D versus dose at lower doses followed by a plateau.^(^ ^57^ ^)^ Evidence suggests that the shape of the dose‐response curve depends on the individual's initial vitamin D status, with lower starting concentrations resulting in the greatest dose response.^(^ ^58^, ^59^, ^60^ ^)^ The plateau in the dose‐response curve only manifests in individuals with preexposure 25(OH)D concentrations of >50 nmol/L.^(^ ^59^ ^)^

Serum 25(OH)D_3_ response in humans in the laboratory depends on total dose but not the dose rate (ie, irradiance).^(^ ^51^ ^)^ Such reciprocity for a given UVR dose of 300 J/m^2^ was also observed for vitamin D_3_ in pig skin using monochromatic radiation at 292, 296, and 300 nm with irradiances that varied by two orders of magnitude^(^ ^50^ ^)^ (it should be noted that these exposures would be about one MED in human skin^(^ ^17^ ^)^). Thus, equivalent increases in vitamin D_3_ concentration can be achieved with high irradiances of UVB over short periods and lower irradiances over long periods. However, this approach has its limits in sunshine because of changes in vitamin D effective solar‐UVB irradiance during the day.^(^ ^33^ ^)^

Dose responses are harder to determine in field conditions because personal UVR exposure must be measured or estimated in retrospect rather than irradiating participants with predetermined known doses. A study in which electronic UVB dosimeters were worn by 98 Europeans during 1‐week sun and skiing holidays showed a linear dose response when exposed body‐surface area (BSA) was taken into account.^(^ ^60^, ^61^ ^)^ Another study, using personal UVR dosimeters and sun‐exposure diaries in 25 healthy White volunteers in Copenhagen (Denmark; 56^o^N) showed that serum 25(OH)D_3_ at the end of summer and winter was dependent on the UVR dose received during the previous summer.^(^ ^62^ ^)^ A study of 100 people with dark skin in South Africa showed that personal UVB exposure was highly correlated with vitamin D status.^(^ ^63^ ^)^

The difference between the CIE action spectra for erythema and previtamin D_3_ means that dose (SED) necessary for vitamin D synthesis varies with the irradiance spectrum of the source^(^ ^42^, ^64^, ^65^ ^)^ as shown in Table 1. Thus, a given level of vitamin D synthesis/SED for an artificial UVR source cannot be directly used to predict a response from solar UVR.

**Table 1:** Effect of Different Spectra on Vitamin D Production

UVR source	UVB % EEE^a^	Study type	BSA exposed	Dose protocol	Δ25(OH)D_3_/SED (nmol/L)	SED for 1 nmol/L 25(OH)D_3_
Sunlight in March (20–27) in Tenerife (28°N) with max UVI of 9^(^ ^184^ ^)^	72% on March 24 at solar noon	7‐day holiday	85%	SPF‐15 sunscreen with high UVA‐PF transmitting more UVB than below	7.0	0.14
				SPF‐15 sunscreen with low UVA‐PF transmitting less UVB than above	4.8	0.21
TL01_(_ _142__)_	99%	Lab study	85%	1.5 SED × 5 Every 3–4 days	7.9 in FST II^b^ 5.5 in FST VI^b^	0.13 0.18
Fluorescent SSR_(_ _142__)_	78%	Lab study	85%	2 SED × 5 every 3–4 days	5.0 in FST II^b^ 4.1 in FST III^b^ 3.9 in FST IV^b^ 4.2 in FST V^b^ 3.9 in FST VI^b^	0.20 0.24 0.26 0.24 0.26
UV6^c^	96%	Lab study	85%	2 SED × 5 every 3–4 days	4.97 in FST I/II^c^	0.20
Fluorescent SSR^c^	80%	Lab study	85%	2 SED × 5 every 3–4 days	3.18 in FST I/II^c^	0.31
PUVA^c^	45%	Lab study	85%	2 SED × 5 every 3–4 days	1.99 in FST I/II^c^	0.50
UV6^c^	96%	Lab study	4%	2 SED × 5 every 3–4 days	1.35 in FST I/II^c^	0.74
SSR^c^ (Xe arc, high UVB)	79%	Lab study	4%	2 SED × 5 every 3–4 days	0.96 in FST I/II^c^	1.04
SSR^c^ (Xe arc, low UVB)	48%	Lab study	4%	2 SED × 5 every 3–4 days	0.55 in FST I/II^c^	1.82
Fluorescent SSR_(_ _145__)_	Unknown	Lab study	35%	Single exposure of 0.2, 0.4, 0.6, & 0.8 MED with each exposure a month apart	3.8 in FST I^d^ 3.1 in FST II^d^ 2.5 in FST III^d^ 1.4 in FST IV^d^ 1.1 in FST V^d^ 0.5 in FST VI^d^	0.26 0.32 0.40 0.71 0.91 2.00

The estimated vitamin D effectiveness per SED varies with the UVR emission spectrum and decreases with decreased UVB EEE for a given BSA exposed. 25(OH)D_3_ = 25‐hydroxyvitamin D_3_; BSA = body‐surface area; EEE = erythemally effective energy; FST = Fitzpatrick skin type; PUVA = psoralen (P) and long‐wave ultraviolet radiation (UVA); SED = standard erythemal dose; SPF = sun‐protection factor; SSR = solar‐simulated radiation; UVA = ultraviolet A; UVA‐PF = UVA protection factor; UVB = ultraviolet B; UVI = ultraviolet index; UVR = ultraviolet radiation; Xe = xenon.

^a^ EEE is the erythemally effective energy for UVB (280–315 nm)—the UVB % contribution to erythema.

^b^ Based on linear regressions [SED vs 25(OH)D_3_] after adjustment for baseline 25(OH)D_3_.

^c^ Article submitted and data based on linear regression [SED vs 25(OH)D_3_] without adjustment for baseline 25(OH)D_3_.

^d^ Personal communication from Professor Lesley Rhodes. Note: all studies are from the same group apart from the last row.

## Atmospheric, Geographic, and Climatic Factors

### Atmosphere

The stratospheric ozone (O_3_) layer attenuates UVB radiation. For any given solar elevation, the O_3_‐layer absorption is the main determinant of environmentally available surface UVB in cloud‐free and low‐aerosol conditions.^(^ ^66^, ^67^ ^)^ Depletion of the O_3_ layer by ozone‐depleting substances (ODSs) from 1980 to 2000 resulted in greater potential exposure to UVB with possible consequent health effects, though changes were small outside the polar regions.^(^ ^68^ ^)^ By phasing out the production of ODSs, the implementation of the Montreal Protocol^1^ 1 will lead to the recovery of the ozone layer. The Montreal Protocol has already prevented a 20% increase in the UVI at midlatitudes between the early 1990s and today.^(^ ^69^ ^)^

The solar zenith angle (SZA) is the angle between the local vertical and the position of the sun. A small SZA provides a short path length for UVR through the atmosphere and low attenuation, whereas a larger SZA increases path length with greater attenuation. Small SZAs (approaching zero) occur only within the tropics near solar noon. At higher latitudes, where solar elevation angles are lower, the minimum SZAs are larger. For example, at latitude 45° the noon‐time SZA ranges from 21.6° in summer to 68.4° in winter. In polar regions (latitudes >66.6°), the sun remains below the horizon (SZA >90°) in the winter months; even in summer, the SZA is always >43.1° at the Arctic Circle. Terrestrial UVR is also modified by atmospheric aerosols and particulates, clouds, and surface albedo (reflectivity). Solar radiation is scattered by air and atmospheric particles in a strongly wavelength‐dependent manner. UVB is much more strongly attenuated than UVA with a greater effect when the path length is longer, such as in winter, at dawn and dusk, and at high latitudes.^(^ ^66^ ^)^

Clouds also attenuate UVR in a wavelength‐dependent way.^(^ ^41^ ^)^ Their modifying properties depend on size, depth, and composition. Although clouds influence visible light more than UVR, their attenuation of UVR contributes substantially to a reduction of UVI. In an urban location in Brazil (19.9°S), mean summer noon UVI values of a minimum of 3 and a maximum of 10 were recorded in overcast conditions,^(^ ^70^ ^)^ whereas with clear skies maximal UVI recordings in the same area were approximately 13. Reduced UVI will inevitably affect vitamin D synthesis.

Paradoxically, if the sun is not obscured, clouds can also enhance terrestrial UVB because of greater forward scattering compared with blue sky. This enhancement has been reported to be from 20%^(^ ^41^ ^)^ to 22% for erythemal UV irradiance.^(^ ^67^ ^)^

The underlying surface reflectivity also affects how much UVR arrives, though very few natural surfaces reflect significant amounts of UVB radiation. The surface albedo is the ratio of reflected‐to‐incident radiation. Snow and ice—and to a lesser extent sand—have high UVB surface albedos.^(^ ^71^, ^72^ ^)^ Fresh snow in unpolluted areas can have a UVR albedo of approximately 98%.^(^ ^73^ ^)^ Such surfaces can contribute to an overall increase in UVB irradiance by reflecting radiation upwards from the Earth's surface, which is then scattered back downward by air molecules, aerosols, and cloud droplets.^(^ ^41^, ^74^ ^)^

### Altitude

Altitude reduces UVB atmospheric path length and increases spectral irradiance as a function of SZA and wavelength.^(^ ^75^, ^76^ ^)^ For example, measurements at a valley and adjacent mountain top in Germany showed 1 km of height increased irradiance at 300 nm by 24%.^(^ ^77^ ^)^ The main contributors to lower irradiance in the valley were ozone absorption and Rayleigh scattering (ie, by atmospheric particles much smaller than the wavelength in question). The effect of altitude on vitamin D photosynthesis has not been extensively investigated. About a fourfold increase in previtamin D_3_ production from 7‐DHC occurs in vitro at Mount Everest in the Himalayas (China and Nepal) base camp (5300 m) as opposed to Agra, India (170 m; the 24^th^ most‐populous city in India)^(^ ^78^ ^)^ but such measurements may be influenced by ground‐level pollution.^(^ ^75^ ^)^ Animal studies at altitudes of 2000 to 2600 m show a higher serum 25(OH)D_3_ in sheep^(^ ^79^, ^80^ ^)^ but not goats.^(^ ^80^ ^)^ In a cohort of 73 patients with ankylosing spondylitis, a 3‐week April holiday in Bad Gastein, Austria (1000 m) significantly increased serum 25(OH)D.^(^ ^81^ ^)^ A cross‐sectional study of 372 Argentinian children living at two different altitudes, 1400 m and 3750 m, showed significant and direct association of vitamin D with altitude.^(^ ^82^ ^)^ However, vitamin D deficiency is found at high altitudes.^(^ ^83^, ^84^ ^)^ Nine climbers on a 2‐week mountaineering expedition at 3200 to 4000 m showed a significant decrease in vitamin D status presumably because of their heavy clothing.^(^ ^85^ ^)^

Confounding factors must be considered. A study of 236 Bolivian children in lowlands (650 m) and highlands (4000 m), who had poor hygiene and nourishment, as well as endemic infections, found a slightly higher prevalence of vitamin D deficiency in the highlands group; the proportion in both groups was approximately 60%.^(^ ^86^ ^)^ A sample of 1222 children from two locations above 1000 m in Himachal Pradesh, India, showed a deficiency of approximately 80%.^(^ ^87^ ^)^

### Latitude

Higher latitudes are associated with larger SZAs that result in lower total UVB irradiances but with an increased diffuse component caused by UVR scattering and decreased vitamin D‐effective UVB. A modeling study across Europe (35°N–69°N) showed vitamin D “winters” of 2 to 8 months from 37°N to 69°N.^(^ ^40^ ^)^ This approach has meant that latitude has been used as a surrogate for vitamin D status in population studies. For example, possible vitamin D deficiency has been linked to mortality from the SARS‐CoV‐2 virus based on the observation that death rate varies with latitude and is very low <35°N where there is no vitamin D winter.^(^ ^88^ ^)^

A study in Australian adults showed that increasing latitude south significantly decreased vitamin D status with a change of 2.28 nmol/L per degree of latitude.^(^ ^89^ ^)^ There are however caveats for the use of latitude as a surrogate for vitamin D status.^(^ ^90^ ^)^ A meta‐analysis of 394 studies investigating global vitamin D status observed no relationship over a wide latitude range after adjustment for age, gender, and ethnicity.^(^ ^91^ ^)^ However, a crude analysis showed a latitude effect for Whites but not for non‐Whites. A study of seven US locations (18°N–44°N) showed no variation in incident vitamin D‐effective UVR with increasing latitude during summer (March–October) but a significant latitude effect was seen in winter (November–February).^(^ ^90^ ^)^ A study in northern Sweden showed that 79.2% of adults (*n* = 1622) had serum 25(OH)D ≥50 nmol/L (January–May) while living above 63°N.^(^ ^92^ ^)^ This was probably caused by a high consumption of dietary vitamin D, supplementation, and sun holidays. Higher serum 25(OH)D concentrations were seen in children in northern Sweden (63°N) than in southern Sweden (55°N). The northern group had a greater intake of vitamin D supplementation.^(^ ^93^ ^)^

Failure to identify a correlation between vitamin D status and latitude is likely based on multiple factors. Dietary habits, sun avoidance behavior, and clothing worn vary considerably throughout the globe. Participants in nutritional studies may be more concerned with their health and vitamin D status; therefore, they are not representative. There is also a lack of standardization of 25(OH)D measurements. Studies on more homogenous populations, which would correct somewhat for these variabilities, have shown an influence of latitude.^(^ ^94^, ^95^ ^)^ Other environmental features that influence vitamin D photosynthesis vary by geographical location. Pollution levels and the architecture of urban spaces both influence UVB insolation.^(^ ^96^ ^)^ Similarly, living in closer proximity to the coast has also been shown to associate with higher vitamin D‐effective irradiance and vitamin D levels.^(^ ^97^ ^)^ Therefore, the use of latitude as a surrogate for vitamin D status should be undertaken cautiously, with consideration of the potentially confounding behavioral, cultural, and environmental factors.

### Temporal factors: season and time of day

**Fig 4:**
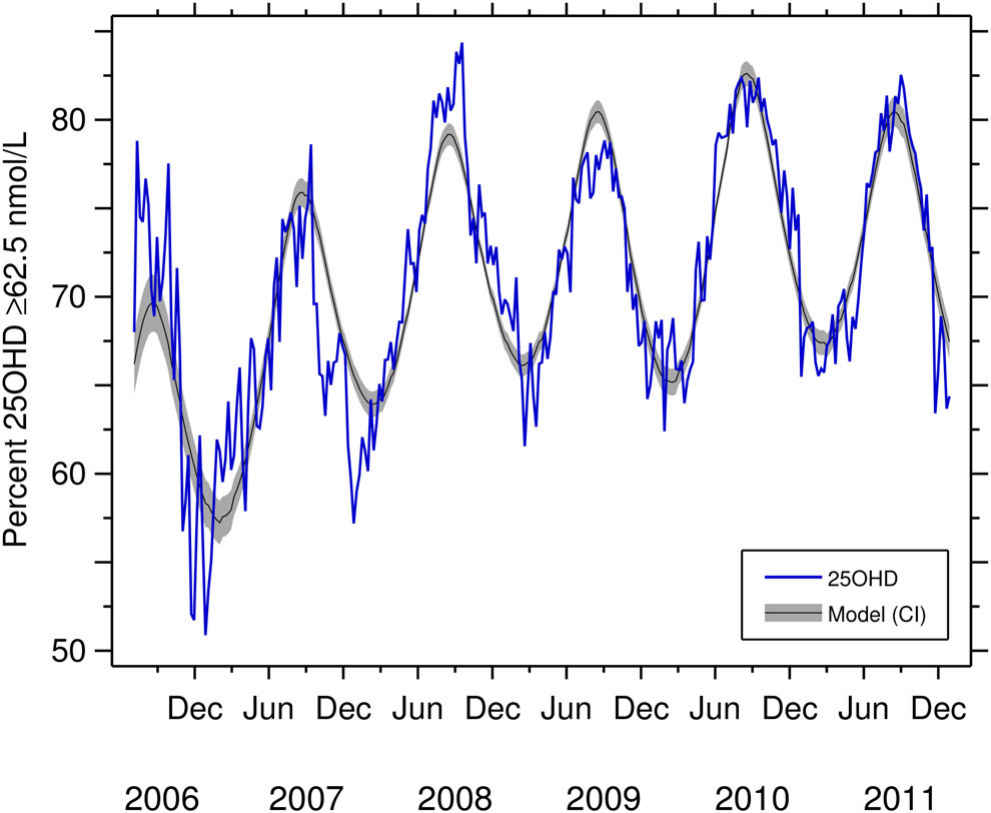
Effect of season on vitamin D status in the United States with serum threshold set at serum 62.5 nmol/L 25(OH)D. The blue line shows data from 3,440,710 individual serum samples from all over the United States that were analyzed by the Mayo Clinic (Rochester, Minnesota, USA), which is a reference laboratory. The black line represents modeled data; the gray band is the 95% CI. Data modified from Fig. 2, Kasahara and colleagues (2013)^(^ ^107^ ^)^ with kind consent from senior author Dr Andrew Noymer. 25(OH)D/25OHD = 25‐hydroxyvitamin D.

SZA is dependent on season and time of day. Studies across a wide demographic, at multiple geographic locations, have reported seasonal variation in vitamin D status,^(^ ^40^, ^98^, ^99^, ^100^, ^101^, ^102^, ^103^, ^104^, ^105^ ^)^ particularly at higher latitudes where seasonal variations in UVB are large. This seasonality is also apparent in in tropical Brazil,^(^ ^106^ ^)^ where seasonal changes in cloud cover are probably important, and in those with dark skins in South Africa.^(^ ^63^ ^)^ At midlatitudes in both hemispheres (eg, the United States^(^ ^107^ ^)^ and New Zealand^(^ ^108^ ^)^), serum 25(OH)D is maximal in late summer and minimal at the end of winter. US data are shown in Figure 4.^(^ ^107^ ^)^ It should be noted that sun avoidance based on excessive heat may reverse this trend. A study of Saudi Arabs (24°N) with high BMI (BMI >25) showed significantly higher serum 25(OH)D in winter compared with summer.^(^ ^109^ ^)^

An in vitro study in Saudi Arabia (24°N) showed that maximal conversion of 7‐DHC to previtamin D_3_ was at 11.00 to 12.00 in summer and winter.^(^ ^110^ ^)^ The summer % conversion at approximately 8% was twice that of winter. Peak synthesis at about this time also provides a better risk‐versus‐benefit ratio as shown by one theoretical study comparing erythema versus previtamin D_3_ synthesis in the absence of sunscreen.^(^ ^111^ ^)^

## Personal Factors

### Skin pigmentation

Skin color, which is dependent on melanin concentration, is phenotypically characterized by a Fitzpatrick skin type (FST) ranging from I (eg, Celtic) to VI (eg, African).^(^ ^112^ ^)^ Melanin, a chromophore that competes with 7‐DHC for UVB absorption, is concentrated in the basal layer of the epidermis.^(^ ^113^ ^)^ Pigmentation may be constitutive (ie, genetic) or facultative (ie, tanning acquired by sun exposure).

There is a melanin gradient with terrestrial UVB in indigenous populations. Melanin decreased as humans in prehistory dispersed away from tropical locations. One driving factor for this loss is thought to be the need to maintain adequate vitamin D synthesis.^(^ ^114^, ^115^ ^)^ However, this view has recently been challenged.^(^ ^116^ ^)^ Mass migration and travel in relatively recent history has meant that people's skin color no longer necessarily matches the solar conditions under which it evolved.

A number of epidemiological studies have reported lower vitamin D status within a given latitude zone in individuals with darker skin types relative to those with lighter skins.^(^ ^117^, ^118^, ^119^, ^120^, ^121^ ^)^ Studies over long periods in the United States show that Black Americans have poorer vitamin D status than their White compatriots.^(^ ^122^ ^)^ This is also true of children living in north and south Sweden.^(^ ^93^ ^)^ Variation in given regions also exists within darker FSTs, with significantly higher increases in serum 25(OH)D in Indian children with FST IV (light brown) skin versus V (dark brown) skin^(^ ^123^ ^)^ when BSA exposed and times outdoors were similar. Limited data suggest that vitamin D status is poor in Africa.^(^ ^26^ ^)^ However, studies of traditionally living East Africans with FST VI found they had serum 25(OH)D >100 nmol/L that the authors attributed to solar UVR rather than diet.^(^ ^124^, ^125^ ^)^ A study of healthy young South Africans with dark and lighter (mixed ancestry) skins showed more 25(OH)D in those with dark skin in summer (median 72.6 vs 65.5 nmol/L).^(^ ^63^ ^)^ This was possibly caused by more time outdoors and BSA exposed. One study in Nigeria compared 25(OH)D status in people with normal pigment with albinos who had similar sun‐exposure patterns. The serum values were high in all cases but were only 23% higher in the albinos (median 95.9 vs 78.25 nmol/L).^(^ ^126^ ^)^ It is difficult to interpret the protective effect of melanin without knowing when the dose‐response curves reach their plateau.

The impact of skin pigmentation, whether constitutive or facultative, on vitamin D photosynthesis is important for accurate public health messages. Facultative face pigmentation changes in FSTs I to III predicted seasonal variation in vitamin D status in a study in Scotland,^(^ ^127^ ^)^ but we lack data on the effect of tanning. Laboratory investigations of the effect of constitutive pigmentation on vitamin D production in response to controlled UVR exposure have yielded conflicting results.^(^ ^128^ ^)^ Some studies have reported that melanin inhibited vitamin D production^(^ ^56^, ^129^, ^130^, ^131^, ^132^, ^133^, ^134^ ^)^ whereas others did not.^(^ ^58^, ^127^, ^135^, ^136^, ^137^, ^138^, ^139^ ^)^ One laboratory intervention study found that variations in pigment single‐nucleotide polymorphisms showed a better relationship with vitamin D response than constitutive and facultative pigmentation.^(^ ^21^ ^)^

A systematic review concluded that studies reporting an inhibitory effect of melanin were more convincing than those that observed no influence but that insufficient evidence was available on the efficacy of vitamin D production in different skin types.^(^ ^140^ ^)^ An observational study in New Zealand showed similar dose‐response slopes for 25(OH)D versus SED for those of European, Maori, Pacific island, or Asian origin (FSTs I‐IV), suggesting no role for melanin.^(^ ^57^ ^)^ It should be noted that this study assumed linearity with BSA exposed. A recent study in France (*n* = 1191) reported that sun exposure and latitude were more important to vitamin D status than the FST.^(^ ^141^ ^)^ Unlike other studies, this study found better vitamin D status with a higher FST. Another recent study in FSTs II‐VI compared increases in serum 25(OH)D_3_ after repeated exposures to the same doses of SSR that were suberythemal in FST II.^(^ ^142^ ^)^ A melanin inhibition factor of 1.3 was obtained by comparing FSTs II and VI. This seems modest but may be enough to explain the epidemiological data. In contrast, the protection factor of melanin against basal layer DNA photodamage is about 60.^(^ ^113^ ^)^ The high concentration of melanin in the basal layer spares nuclear DNA (a major chromophore) but there is ample 7‐DHC above the basal epidermis^(^ ^143^, ^144^ ^)^ which has much less melanin even in FST VI. However, another study reported that the vitamin D responses were similar in different FSTs (I‐VI) when doses of SSR were given as a function of MED.^(^ ^145^ ^)^ Thus, a comparison of vitamin D synthesis in FST I and FST VI in this study would indicate that the inhibition factor afforded by melanin was in the region of eight (see Table 1).

### Sun behavior

Season and latitude may be poor markers of vitamin D because of confounding behavioral factors.^(^ ^146^ ^)^ Low supplement use, poor dietary intake of vitamin D, and sun‐avoidance behaviors are associated with low vitamin D status.^(^ ^147^, ^148^, ^149^ ^)^ Despite any effects of latitude, certain populations in northern Europe have better vitamin D status than populations in southern Europe^(^ ^150^ ^)^ because of vitamin D‐rich diets.^(^ ^104^ ^)^

People from mid to high latitudes often take sun holidays that can result in a greater summer vitamin D peak.^(^ ^105^, ^127^ ^)^ A sun holiday in 2019 increased winter 25(OH)D by 20 to 30 nmol/L in native and immigrant Swedes from Uppsala (60°N).^(^ ^151^ ^)^ Similarly, summer sun holidays were shown to improve vitamin D status in postmenopausal women in Aberdeen, Scotland (57°N).^(^ ^99^ ^)^ Summer holidays abroad by people living in Orkney (57°N –59°N), an archipelago in northern Scotland, may account for better vitamin D status than mainland Scotland.^(^ ^152^ ^)^ Summer and winter serum 25(OH)D were greater in UK adolescents who had taken a holiday; it accounted for a 17% variation in peak vitamin D status.^(^ ^105^ ^)^ A beach holiday in the previous year predicted 6.4% of the variance in vitamin D levels in an Italian cohort of 620 participants.^(^ ^153^ ^)^

However, sun holidays result in short periods of intense UVR exposure, which has been shown to associate with skin cancer at all latitudes.^(^ ^60^ ^)^ Petersen and colleagues found a strong correlation between holiday UVB exposure, positive vitamin D response, and an increase in DNA damage over a 6‐day March holiday.^(^ ^60^ ^)^ It should be noted that the Danes who participated in the holiday study (in Tenerife in the Canary Islands, Spain) had 43% of the annual UVR burden of a Danish indoor worker.^(^ ^154^ ^)^ A 12‐day summer holiday taken by Polish children on the Baltic Sea with a modest daily‐exposure dose of 2.4 SED (without taking any effect of sunscreen into account) showed a change from 64.7 to 79.3 in nmol/L serum 25(OH)D_3_ (mean increase of 14.7 ± 12.4),^(^ ^155^ ^)^ which had fallen to 68.2 nmol/L in October. The modest summer increase, perhaps because of a high baseline, was accompanied by a 12.6‐fold increase in DNA damage. Further investigation of the risks and benefits of holiday UVR exposure is required.

Clothing plays a determinant role in vitamin D status. This is affected by many complex interactions: For example, among Austrian women increases in temperature led to less body coverage but also to less time outdoors.^(^ ^156^ ^)^ Cultural factors are also important. Vitamin D deficiency was found to be prevalent in Kuwaiti women wearing Western and traditional clothing but was more marked in the latter group.^(^ ^157^ ^)^ Similarly, hijab‐wearing women in Nova‐Scotia (Canada) had less good vitamin D status than Western‐dressing counterparts.^(^ ^158^ ^)^ Another factor to consider is physical activity, which may contribute to better vitamin D status independently of sunlight and dietary intake.^(^ ^159^ ^)^ In addition, poor public knowledge of vitamin D may contribute to low prevalence of vitamin D supplementation and therefore lower vitamin D status.^(^ ^160^ ^)^

### Body site and surface area exposed

Knowledge of the relationship between UVR dose, body site, and BSA irradiated, and vitamin D response is important for accurate public health messages.^(^ ^68^ ^)^ This relationship has been investigated in vivo under laboratory conditions by exposing variable proportions of BSA to UVR.^(^ ^161^, ^162^, ^163^, ^164^, ^165^, ^166^ ^)^ In general, a positive correlation has been observed between BSA exposed and vitamin D response with some variation depending on anatomical location.

A winter study in Finland exposed different groups of FST II and FST III women to seven consecutive daily exposures (1 SED 1st day and 2 SEDs thereafter) of narrow band UVB to different body areas. The increase in the group with abdominal exposure was 4 nmol/L. Increases of approximately 11 nmol/L 25(OH)D were observed in groups that either had full‐body or head plus arms exposures. The same protocol to face plus arms group with SSR showed a smaller increase of 3.8 nmol/L.^(^ ^162^ ^)^ Increasing BSA exposed resulted in better end‐of‐summer vitamin D status in a study of office workers in Australia with values of approximately 47 and approximately 89 nmol/L of serum 25(OH)D for face plus hands and whole‐body exposure, respectively,^(^ ^167^ ^)^ suggesting a nonlinear relationship.

**Fig 5:**
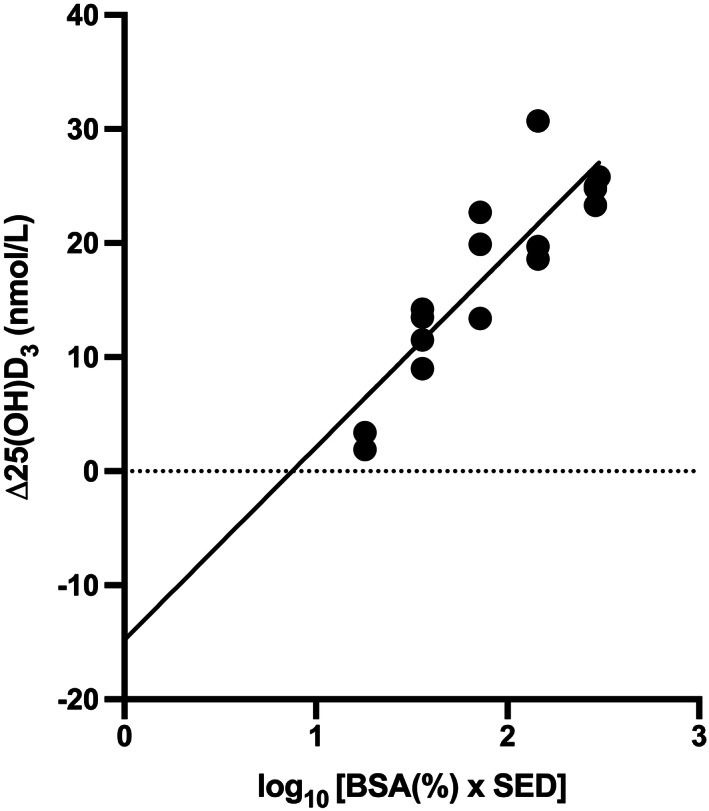
Linear relationship between increase of 25(OH)D_3_ with log_10_ product of total exposure dose (SED) and BSA exposed (6, 12, and 24%–25%). Regression equation; y = 16.89x – 14.80, *R* ^2^ = 0.7739, *p* < 0.0001. Each volunteer was exposed to four equal doses (0.375–3.0 SED) of a broadband ultraviolet B phototherapy source. Data taken from several studies from the same laboratory.^(^ ^51^, ^58^, ^64^, ^164^, ^198^ ^)^ 25(OH)D_3_ = 25‐hydroxyvitamin D_3_; BSA = body‐surface area; SED = standard erythemal dose.

Increasing BSA (6%, 12%, and 24%) showed a linear relationship with increased serum 25(OH)D after four exposures of 0.75 SED broadband UVB but not with 1.5 and 3 SED.^(^ ^164^ ^)^ Dose‐response analyses in the same study showed significant linear responses at 6% and 12% BSA but no effect at 24% BSA. These results suggest a complex relationship between UVB dose and BSA exposed. Figure 5 suggests a linear relationship between increase in 25(OH)D_3_ and the log_10_ product of SED and BSA exposed when BSA is between 6% to 25%. Small but nonsignificant increases in vitamin D response have been seen with increased BSA (15 vs 30%) exposure to sunlight in children in India with FST IV and FST V skin.^(^ ^123^ ^)^ Figure 3 shows the UVR dose–response curves for vitamin D with 3.7% (defined area on trunk) and 85% BSA exposure (underwear only). It can be seen that a greater than 20‐fold increase in BSA results in a 3.7‐fold increase in the rate of 25(OH)D_3_ production, suggesting a positive but nonproportional relationship between BSA and vitamin D photosynthesis. Therefore, a comparison of Figs. 3 and 5 suggests a failure of reciprocity for UVR dose × BSA exposed when the BSA are extremes. This also suggests a homeostatic process that limits systemic vitamin D toxicity. CYP2R1 is the gene that codes for the enzyme that converts vitamin D to 25(OH)D. Exposure of FST I/II skin to 6 SED SSR reduced cutaneous CYP2R1 mRNA expression (at 6 and 24 hours postexposure), which also suggests a local feedback mechanism.^(^ ^168^ ^)^

Factors other than BSA are likely to be important. 7‐DHC concentration may vary with body site. For example, a study in chickens reported that its concentration in leg and feet skin was 30 times greater than the back.^(^ ^169^ ^)^ Consequently, whole‐body exposure with a given UVB dose to defeathered birds resulted in previtamin D photosynthesis in the legs and feet but not in the skin on the back.

In humans, UVR transmission varies with body site, and this will impact on vitamin D synthesis.^(^ ^33^ ^)^ A significantly steeper vitamin D_3_ (cholecalciferol) response was produced in the upper body and full body compared to the hands plus face^(^ ^166^ ^)^ (data from acute erythemal and repeated sun‐erythemal broadband UVB exposures combined). In another study, in which different body sites were differentiated by sunscreen protection, significant increases in serum vitamin D_3_ (cholecalciferol) were seen in trunk, legs, and whole body of participants after a single suberythemal UVR dose (260–360 nm), but not for the arms or head plus neck.^(^ ^161^ ^)^ Another study with three consecutive suberythemal broadband UVB exposures showed different results with serum 25(OH)D_3_ and vitamin D_3_. Whole‐body exposure gave the same results as the face plus hands with 25(OH)D_3_ but was considerably more effective than face and hands for vitamin D_3_.^(^ ^163^ ^)^

### Sunscreen use

Sunscreen efficacy is measured by sun‐protection factor (SPF). This is the ratio of MED with and without sunscreen applied under laboratory conditions using SSR.^(^ ^170^ ^)^ Erythema is primarily induced by UVB (Fig. 1), making SPF mainly, but not exclusively, a measure of UVB protection.

The SPF is calculated at 2 mg/cm^2^ but users typically apply much less^(^ ^171^ ^)^ (eg, 0.79 mg/cm^2^ by Danes on holiday in Egypt^(^ ^172^ ^)^), and so receive suboptimal protection.^(^ ^172^, ^173^, ^174^ ^)^ Application of an SPF 50 sunscreen at 0.75 mg/cm^2^ will provide a real SPF of approximately 20.^(^ ^175^ ^)^

The overlap of the erythema and previtamin D_3_ action spectra in the UVB region, suggests that sunscreens block vitamin D photosynthesis (Fig. 1). Indeed, sunscreens and other types of photoprotection have been shown to associate with low vitamin D status.^(^ ^161^, ^176^, ^177^, ^178^, ^179^ ^)^ Some laboratory studies have used inappropriate nonsolar UVB sources and their conclusions can be considered invalid.^(^ ^23^ ^)^ In a study of 5920 adults, seeking shade and wearing long‐sleeved clothing was associated with lower 25(OH)D levels but frequent low, moderate, or high sunscreen use had no effect.^(^ ^180^ ^)^ Reviews on sunscreens and vitamin D status have concluded that typical use has little or no impact.^(^ ^23^, ^128^, ^181^, ^182^ ^)^ However, sunscreen use in Australia may be associated with higher vitamin D status because people spend more time outdoors.^(^ ^167^ ^)^ Sunscreen use in a study in Scotland also resulted in better vitamin D status.^(^ ^127^ ^)^ A field study in Tenerife compared discretionary sunscreen use with interventional guided optimal use.^(^ ^183^, ^184^ ^)^ Sunburn was observed in the former but not in the latter group. Vitamin D status improved significantly in both groups, though the increase was greater in the group with sunburn. The reason for the modest effect of sunscreens is that the dose threshold for vitamin D synthesis is much lower than that for erythema.

The absorption spectra of sunscreens depend on their mix of active ingredients. It has been suggested that based on the CIE action spectrum for previtamin D_3_, their absorption spectra can be tailored to maximize vitamin D synthesis.^(^ ^185^ ^)^ There were two intervention SPF = 15 sunscreens in the Tenerife study referred to above.^(^ ^184^ ^)^ However, one transmitted more UVB and enabled significantly more vitamin D synthesis (Table 1).

## How Much Sunlight Is Necessary to Maintain Optimal Vitamin D Status?

There are different methods to determine the minimal amount of solar UVB necessary for maintaining vitamin D status. One approach is climatic modeling using the previtamin D_3_ action spectrum.^(^ ^186^ ^)^ This depends on the accuracy of this spectrum for serum 25(OH)D_3_ and assumes no spectral interactions ( eg, significant photodegradation by UVA). Many studies have used UVB phototherapy sources but these are likely to overestimate vitamin D production for a given erythemal exposure protocol (see Table 1). The use of SSR sources is likely to give a better estimation. The ideal approach is sunlight in which change of vitamin D status is compared with personal exposure and BSA exposed.

The number of physical variables that affect vitamin D status makes it hard to give precise UVR exposure recommendations for optimal vitamin D status. Studies use different designs, assumptions, and target endpoints. This is further complicated by biological (eg, genetics^(^ ^187^ ^)^) and clinical factors (eg, BMI^(^ ^188^ ^)^ and disorders^(^ ^23^ ^)^). Given seasonal variation, it is also important to generate sufficient vitamin D in summer to provide winter reserves.

Vitamin D status can be maintained above 50 nmol/L throughout the UK winter if sufficient stores are generated during the warm months but the majority of the population fails to do so.^(^ ^100^ ^)^ Increasing daily summer sun exposure has been reported to have minimal effect on winter vitamin D levels and may increase the risk of the negative consequences of UVR exposure.^(^ ^189^, ^190^ ^)^ A large New Zealand observational study suggests regular exposures of <2 SED solar UVR/week (adjusted for BSA exposed) is sufficient for good vitamin synthesis^(^ ^57^ ^)^ but increasing the weekly exposure dose brought diminishing returns.

Using a UK climatic model, it has been estimated that 1 SED daily at lunchtime for those with light skins (9–13 minutes of exposure depending on latitude) is sufficient from March to September in season‐appropriate clothing to achieve an end‐of‐summer target of 80.5 nmol/L of 25(OH)D to maintain a winter vitamin D status of 25 nmol/L.^(^ ^186^ ^)^ A similar analysis for the same winter target for FST V showed a daily dose of 2.75 SED (25–40 minutes) is needed to achieve an end‐of ‐summer target of 85.8 nmol/L 25(OH)D.^(^ ^191^ ^)^ In all cases, approximately 35% BSA (eg, forearms and lower legs) would need to be exposed between June and August.

One study in central England exposed 35% BSA of FST V with low baseline 25(OH)D to different SSR doses (0.65–3.9 SED) 3 times per week for 6 weeks. In general, there was a dose‐dependent effect, though the best response was with 3.25 rather than 3.9 SED. However, in no case did mean serum 25(OH)D reach 50 nmol/L.^(^ ^56^ ^)^

A study of Danish girls and older women suggested a summer peak of 100 nmol/L was necessary to obtain a winter level of 50 nmol/L.^(^ ^98^ ^)^ This is similar to results from data and modeling for central England.^(^ ^192^ ^)^ These data showed serum 25(OH)D <50 nmol/L from November to May. Modeling estimated that daily oral intake of 3 μg plus 2 hours local sun exposure on weekdays and 3 hours on weekend days on unprotected skin with a maximum of 20% BSA exposed would increase the summer peak to >100 nmol/L and February nadir from approximately 38 to 58 nmol/L. In contrast, the same dietary intake with weekday and weekend times of 30 and 45 minutes, respectively, would not result in 50 nmol/L at any time. A study of indoor workers in Sydney, Australia (33.9°S) showed a significant linear relationship between end‐of‐summer and end‐of‐winter serum 25(OH)D.^(^ ^167^ ^)^ An end of summer value of approximately 60 nmol/L was associated with an end‐of‐winter status of 50 nmol/L, which suggests that some vitamin D synthesis occurred in autumn/winter.

Sun holiday studies,^(^ ^60^, ^184^ ^)^ in which typically a very high BSA is exposed, have shown a significant increase in vitamin D status that is accompanied by sunburn: i.e., excess UVB. In one study, in Tenerife in March, some participants were given sunscreen with SPF‐15 and instructions on optimal use.^(^ ^184^ ^)^ Participants did not sunburn, and they showed a highly significant improvement in vitamin D status over 1 week. Personal exposure was measured as SED. It is therefore possible to estimate erythemal dose to the skin through the sunscreen and equate this with change in vitamin D status. This was approximately 0.4 SED/day through the sunscreen in the study described above,^(^ ^184^ ^)^ which equates to about 0.13 MED in a FST II person.^(^ ^16^ ^)^ This resulted in an increase of 4.8 to 7.0 nmol/L 25(OH)D_3_ per SED, depending on the optical properties of the sunscreen (Table 1). An important advantage of repeated low‐dose exposure (eg, 1.3 SED SSR thrice weekly) to maintain vitamin D status is a reduction in DNA damage.^(^ ^193^ ^)^

Given the uncertainties of the vitamin D action spectrum, the effect of BSA exposed, and the role of melanin, it is difficult to give precise exposure recommendations. However, it is important to note that considerable vitamin D synthesis occurs with suberythemal exposures.

The most prudent advice may be to encourage suberythemal sun exposure during the summer and autumn and increase dietary intake and supplementation during the winter.^(^ ^108^, ^190^ ^)^

## Conclusions

Multiple factors influence the quantity and quality of solar UVB reaching the skin for vitamin D photosynthesis. The effect of quality of UVR in terms vitamin D efficacy per SED is shown in Table 1. UVR dose‐response studies show an initial linear response followed by a plateau, possibly because of UVA‐mediated regulatory mechanisms.^(^ ^11^, ^45^ ^)^ Vitamin D production is dependent on dose but not dose rate, which in theory means that longer periods of lower irradiance sun exposure are as effective as and safer than short periods of high‐intensity exposure. However, solar irradiance and emission spectrum varies with SZA, which is constantly changing at a given latitude, so the balance between the effects of exposure time on benefit and risk fluctuates. Public health advice must take these factors into account. BSA is positively correlated with vitamin D response but the relationship is nonlinear. It is likely that production of vitamin D differs depending on anatomical location, owing to varying optical properties of skin and different levels of cutaneous 7‐DHC in different skin layers.^(^ ^144^ ^)^ Furthermore, UVR exposure may alter the skin's UVR transmission properties.^(^ ^194^ ^)^ This may in part explain the nonlinear relationship between BSA and vitamin D response. However, real‐life world data on dose responses are lacking, and further investigation is needed to assess the variability in response between exposure sites.

Direct investigation of the relationship between skin pigmentation and vitamin D response has produced conflicting results.^(^ ^128^ ^)^ The degree of inhibition by melanin on vitamin D photosynthesis requires further investigation in high‐quality studies with large sample sizes in conditions representative of real life.^(^ ^140^ ^)^ The consensus on sunscreen use is that it has limited effects but intervention studies are required at temperate latitudes^(^ ^195^ ^)^ and with high SPF sunscreens. Photoprotection by behavior and clothing has inhibitory effects even in countries with high insolation.

Laboratory studies can give valuable results but the irradiance spectrum in such studies needs careful consideration to avoid misleading conclusions. The action spectrum for vitamin D_3_ production is under debate. An incorrect action spectrum will lead to misleading risk/benefit calculations from solar exposure.

Laboratory and outdoor studies show that repeated suberythemal exposures are sufficient to improve and maintain optimal vitamin D status. This is in line with public health advice to avoid sunburn to minimize skin cancer risk in those with light skins. Supplementation may provide a safer means of obtaining an adequate vitamin D status in those who are suboptimal. However, this approach may avoid benefits of solar exposure that are independent of vitamin D. There are reports of increasing toxicity^(^ ^44^ ^)^ although this is rare and may be caused by manufacturing and labeling errors.^(^ ^196^, ^197^ ^)^

Improved knowledge of the factors that influence the interactions of UVB with cutaneous 7‐DHC will result in better public health messages to maintain safer levels of solar exposure for given populations.

There is a need for a better understanding of vitamin D synthesis in different body sites. Obviously, further investigation is required to clarify whether, and in what contexts, certain anatomical locations are more effective at photosynthesizing vitamin D. That a proportionately greater increase in vitamin D status is achieved with larger BSA exposure—especially at low doses of UVB—remains unquestionable.

## Disclosures

None of the authors has any conflict of interest to declare.

## AUTHOR CONTRIBUTIONS

**Jonathan Neville:** Conceptualization; data curation; investigation; methodology; resources; writing‐original draft. **Tommaso Palmieri:** Data curation; formal analysis; investigation; methodology; writing‐review and editing.

### Peer Review

The peer review history for this article is available at https://publons.com/publon/10.1002/jbm4.10460.

## Footnotes

1
The Montreal Protocol on Substances that Deplete the Ozone Layer is the landmark multilateral environmental agreement that regulates the production and consumption of nearly 100 man‐made chemicals referred to as ozone depleting substances (ODS). When released to the atmosphere, those chemicals damage the stratospheric ozone layer, Earth's protective shield that protects humans and the environment from harmful levels of UVR from the sun. Adopted on 15 September 1987, the Protocol is to date the only UN treaty ever that has been ratified every country on Earth ‐ all 198 UN Member States.
